# Bridging Pixels and Practice: AI-Assisted Mammography as a Next-Generation Diagnostic Strategy for Breast Cancer Screening

**DOI:** 10.3390/diagnostics16060873

**Published:** 2026-03-15

**Authors:** Dulanjani Galappaththi, Malinda De Silva, Muhammad Jawed, Syed M. Shahid

**Affiliations:** 1 Department of Healthcare, New Zealand School of Vocational Education & Training (NZSVET), Auckland 1010, New Zealand; dulanjani@healthcareer.nz; 2 School of Health & Sport Science, Eastern Institute of Technology (EIT), Auckland Campus, Auckland 1010, New Zealand; 3 Health New Zealand, Te Whatu Ora, Auckland 0620, New Zealand; malindade@otahuhuhealth.co.nz; 4 Department of Biochemistry, Fazaia Ruth Pfau Medical College (FRPMC)/Air University, Karachi/Islamabad, Pakistan; drmjawed@yahoo.com

**Keywords:** AI-assisted mammography, breast cancer screening, benefits, diagnostic efficacy, diagnostic accuracy, challenges, attitudes, views, stakeholder perspectives

## Abstract

Breast cancer remains a leading cause of cancer-related mortality among women worldwide. Mammography is the cornerstone of population-based breast cancer screening, significantly improving prognosis and survival outcomes. However, limitations related to diagnostic accuracy, efficacy, and inequitable access persist. Recent advances in artificial intelligence (AI) have transformed AI-assisted mammography into a next-generation diagnostic strategy capable of enhancing screening performance within integrated diagnostic pathways. This narrative review examined whether AI-assisted mammography could serve as an effective next-generation diagnostic approach for breast cancer screening in global healthcare settings. Following PRISMA guidelines, studies were systematically screened using predefined eligibility criteria and quality appraisal with the CASP checklist, resulting in the inclusion of fourteen selected studies for analysis. Thematic synthesis identified three key domains: benefits, challenges, and stakeholder perspectives. AI-assisted mammography demonstrated improved cancer detection rates, enhanced diagnostic accuracy, reduced workload, and reduced recalls. However, challenges related to legal, ethical and social issues as well as transferability remain significant. Stakeholder perspectives emphasised the importance of human oversight, interdisciplinary collaboration, and workforce readiness. In conclusion, AI-assisted mammography offers significant promise as a next-generation diagnostic strategy, but successful translation into clinical practice requires substantial systemic, organisational, and practice-level changes across healthcare settings.

## 1. Introduction

Breast cancer remains the most commonly diagnosed cancer among women worldwide and a leading cause of cancer-related mortality [1]. Global Cancer Observatory data indicate that approximately 2.3 million new cases were diagnosed in 2020 [2], with an estimated 670,000 deaths reported in 2022 [3]. Projections suggest a continued escalation in global burden, with more than three million new diagnoses and over one million deaths anticipated annually in the coming decades [3]. These trends underscore the urgent need to strengthen early detection strategies within population-based screening programmes.

Mammography remains the cornerstone of breast cancer screening, with evidence demonstrating a 20–30% reduction in breast cancer mortality through organised mammography-based programmes [4]. Despite its proven effectiveness, conventional mammographic screening faces persistent challenges, including diagnostic inaccuracies in dense breast tissue, increasing radiologist workload, and inequities in access to skilled interpretation across regions [5]. These limitations have stimulated interest in technological innovations capable of enhancing diagnostic performance and system efficiency.

Artificial intelligence (AI), particularly machine learning and deep learning algorithms, has emerged as a next-generation diagnostic strategy with the potential to transform mammography interpretation. AI-assisted mammography extends beyond image recognition to function as a clinical decision-support tool within the broader diagnostic ecosystem, supporting radiologists in detection, triage, and risk stratification. The unique characteristics of breast imaging large datasets, standardised acquisition, and well-defined diagnostic endpoints make mammography especially amenable to AI integration [5].

While growing evidence suggests that AI-assisted mammography can improve cancer detection rates, improve diagnostic accuracy and efficiency, and alleviate workforce pressures, its successful translation into routine clinical practice requires careful consideration of clinical workflow integration, ethical and legal governance, and economic sustainability. Public trust, transparency, equity, and the preservation of human oversight are particularly salient in screening contexts, where general populations are involved.

This narrative review aims to synthesise clinically relevant global evidence on the effectiveness of AI-assisted mammography, positioning it as a next-generation diagnostic strategy of breast cancer screening. Specifically, the review examines the feasibility of using this strategy in breast cancer screening programmes by exploring different perspectives to enhance screening performance. The focus is deliberately clinical; therefore, bioengineering and algorithm development studies were not prioritised. By adopting a translational lens, this review seeks to inform policymakers, clinicians, and health system leaders on the feasibility and responsible adoption of AI-assisted mammography across diverse healthcare settings.

## 2. Methods

This research adopted a narrative literature review as the study design, while incorporating systematic search approaches to ensure comprehensive coverage of the evidence base. Literature identification, screening, and selection processes were reported in accordance with PRISMA guidelines. The literature search was conducted across three electronic databases: PubMed, ProQuest Central, and ScienceDirect. These databases were selected to prioritise clinically oriented and peer-reviewed medical literature. Engineering-focused databases (e.g., IEEE, Xplore, Scopus) were excluded by design, as the review focused on clinical performance, implementation and stakeholder perspectives rather than algorithmic development. A comprehensive set of keywords was used to capture the evolving scope of AI-enabled breast cancer diagnostics, including AI-driven, AI-assisted mammography, breast cancer screening, mammography, diagnostic efficacy, diagnostic accuracy, translational potential, workflow integration, performance, benefits, challenges, attitudes, views, and perceptions. Boolean operators (“AND”, “OR”, and “NOT”) were applied to construct structured search strings and expand retrieval. The primary search string employed was: (“AI-driven” OR “artificial intelligence” OR “AI-assisted mammography”) AND (“screening” OR “detection” OR “mammography” OR “breast cancer screening”) AND (“efficacy” OR “diagnostic efficacy” OR “performance”) AND (“workflow integration” OR “translational potential”) AND (“views” OR “attitudes” OR “stakeholder perceptions”).

This broad and targeted search strategy with clinically targeted approach enabled the identification of foundational studies, emerging research trends, and stakeholder-focused evidence relevant to the diagnostic, translational, and implementation dimensions of AI-assisted mammography.

### 2.1. Inclusions and Exclusions

To ensure the relevance and quality of selected articles, a strict set of inclusion and exclusion criteria was applied. Only peer-reviewed journal articles published in the last five years (from 2020 onwards) were considered, with a prerequisite for full-text availability in English or with an English translation. The primary focus of the articles needed to be on AI-driven mammography for breast cancer screening or early detection. Conversely, articles published before 2020, those not peer-reviewed, abstract-only articles, or those in languages other than English were excluded. Only primary studies were included; review articles and secondary analyses were intentionally excluded to avoid duplication of existing synthesis. Furthermore, articles focusing on other imaging modalities like Ultrasound Scan (USS) and Magnetic Resonance Imaging (MRI), or those pertaining to breast cancer staging, grading, treatment, and complications, were also omitted to maintain a clear scope. Finally, only primary studies were included, with reviews and all types of secondary studies being excluded.

### 2.2. CASP Checklist

The CASP (Critical Appraisal Skills Programme) checklist was used for evaluating the methodological quality and risk of bias of the studies included. Both qualitative and quantitative CASP tools were applied, reflecting the mixed study designs included in this narrative review. Table 1 presents the Critical Appraisal Skills Programme (CASP) tool questions adapted for both quantitative and qualitative studies, based on the frameworks described by Long and Calvery studies.

### 2.3. PRISMA Guidelines and Selection of Articles

**Figure 1:**
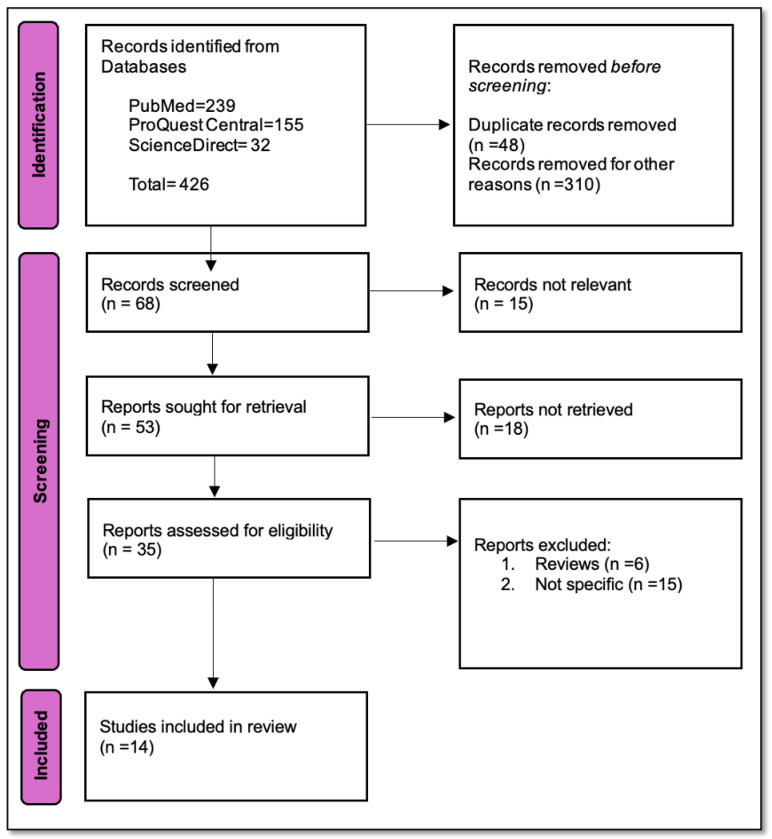
PRISMA flowchart: summary of literature identification, screening, and selection [6].

The article selection process followed the PRISMA 2020 flow diagram, beginning with an initial identification of 426 records from various databases: 239 from PubMed, 155 from ProQuest Central, and 32 from ScienceDirect. After removing 48 duplicate records and an additional 310 records for other reasons (like algorithm development, computational optimisation, non-mammographic imaging modalities like MRI, USS, non-screening applications like diagnosis, staging, non-peer-reviewed articles and review articles or secondary analysis), 68 unique records proceeded to the screening phase. Out of these, 15 were deemed not relevant, leaving 53 reports to be sought for retrieval. Unfortunately, 18 of these reports could not be retrieved, resulting in 35 reports being assessed for eligibility. During this final assessment, 6 reports were excluded as reviews and 15 were deemed not specific to the study’s focus. Ultimately, a total of 14 studies were included in the review as presented in Figure 1.

### 2.4. Summary of Literature

Each selected article was read critically to identify its key findings, theoretical frameworks, methodologies, and conclusions pertinent to the review’s scope. This involved note-taking and conceptual mapping to group similar ideas, identify diverging viewpoints, and discern overarching themes or patterns within the literature. The synthesis then focused on weaving these extracted insights into a coherent narrative, connections, contradictions, and areas where further research is needed, thereby building a comprehensive and interpretive overview of the chosen theme.

Table 2 is introduced here to contextualise the evidence base and summarise key study characteristics, findings and limitations. In this study, 14 articles were reviewed and provided thematic analysis in the field of AI use in mammography for early detection of breast cancer. The thematic analysis revealed three key areas concerning AI-driven mammography in breast cancer screening. Firstly, the benefits were clearly identified, including improved diagnostic accuracy, increased diagnostic efficacy by reduced workload for healthcare professionals, high cancer detection rate, and a reduced breast cancer recall rate for patients. Additionally, technology presents significant opportunities for the detection of interval cancers and for more precise risk assessments. Secondly, the analysis highlighted various challenges associated with AI-driven mammography, notably the ethical, legal and social challenges, as well as implementation challenges. Finally, the study explored the perspectives of both women and professionals regarding the use of AI in mammography, encompassing women’s perceptions and attitudes and the views of professionals.

### 2.5. Findings

The key findings of the articles reviewed in this study are summarized under the following themes and subthemes as provided in Table 3.

#### 2.5.1. Benefits: Improved Cancer Detection Rates, Diagnostic Accuracy and Diagnostic Efficacy

AI-driven mammography gives several benefits for breast cancer screening, which include high early detection rates, increased diagnostic accuracy, improved diagnostic efficacy by reducing workload for radiologists, reduced recall rates and improved interval cancer detection rates on breast cancer screening.

In this review, several studies showed high breast cancer detection rates using AI in mammographic screening. Under the subthemes of Increased Breast Cancer Detection Rates: AI has the potential to increase the cancer detection rates and enhance the capacity of the breast cancer screening programme [8,13]. Breast cancer screening can be enhanced by replacing one radiologist with AI for independent reading, which gives higher cancer detection rate compared to double reading done by two radiologists [8]. Santeramo’s study also reveals increased cancer detection rates by use of AI-driven mammographic screening; in addition, AI enhanced the performance of risk assessment in smaller cancers [19]. Increased Diagnostic Accuracy: A study done by Elhakim has investigated the accuracy of a commercially available AI system for cancer detection. It was found that higher diagnostic accuracy with high sensitivity in AI-integrated screening. It also suggested that replacement of the first reader in double reading by AI system with an appropriate threshold could be feasible [9]. AI could be used as an initial reader, and it could reduce false negative rates in breast cancer screening [15]. AI CAD could improve the specificity of radiologists’ digital mammography interpretation, which could be beneficial for the patient by reducing false positive rates [4]. Increased efficacy by reduced Workload: Several studies have demonstrated the potential of AI-driven mammography to reduce radiologist workload in clinical practice. A study done in Spain found that AI-assisted mammography could reduce the workload up to 70% [18]. A study done in Sweden revealed that the use of AI in mammography reduced screen reading workload by 44% [14]. A Korean study clearly demonstrated how the AI-CAD system improves the performance of radiologists, which would help to reduce their workload [4]. In reduced cancer recall rates: Digital mammography interpretation supported by AI suggests that it could benefit the patients by reducing false positives and reducing cancer recall rates [4]. Several mechanisms contribute to AI’s ability to reduce cancer recall rates, such as improved specificity and standardised interpretations [18]. Using AI in digital mammography screening had a 25% improvement in sensitivity and 27% reduction in breast cancer recall rates [18]. Lower recall rates reduce the healthcare cost related to unnecessary follow-up imaging and minimise the burden on healthcare systems.

In addition to the benefits of AI-driven mammography, increased ‘interval breast cancer’ detection rates were the important opportunities that were identified in this narrative review. According to a study done in Turkey, AI detected an additional 44% of interval cancers which were not previously detected by radiologists [13]. This also reveals that the use of AI can analyse mammograms with higher accuracy than the human eye and can be used effectively in breast cancer screening programmes. AI detection algorithms with high performance for smaller cancers would achieve relatively high performance for risk assessments [19]. Improvement in risk assessment enhances the cancer detection capabilities of smaller cancers, which is an additional advantage.

#### 2.5.2. Challenges: Legal, Ethical, Social, and Implementational Challenges

AI-driven mammography gives many benefits in early detection of breast cancer; however, it also faces several challenges and limitations. One major challenge is the ethical and legal implications. Biases and discrepancies, trust and accountability issues, data privacy and confidentiality are some implications that have been raised recently. Furthermore, social implications like equity and accessibility and cost-effectiveness were other concerns that were faced by AI-driven mammography. Addressing these challenges is crucial to ensure that AI-driven mammography is incorporated into clinical practice safely and effectively. The review acknowledges recent advances in explainability but does not undertake a technical evaluation of explainable AI methods, as this lies outside the clinical scope of the manuscript.

One of the main ethical challenges was the potential for AI algorithm biases and discrepancies. In a study done among Norwegian breast radiologists, it was well highlighted that risk of bias could arise due to the AI algorithms being trained on data [16]. This same study highlighted the discrepancies between the AI systems and radiologists, which need to be addressed. Another ethical challenge identified was the lack of transparency and explainability. Many AI algorithms have a ‘black boxes’ nature, which makes it difficult to understand how they arrive in diagnosis, and this lack of transparency can limit trust when implementing this technology in clinical practice [11]. This transparency issue can raise concerns about trust and accountability. In a study done among Swedish radiologists, the fact about the accountability of AI use was brought up. Who is responsible for the possible errors that could happen when AI is used as a standalone reader? [11]. This could directly affect the trust of the patients towards the healthcare system. In Johansson’s study, participants emphasised transparent screening, and they highlighted that it is important to inform the patients about the use of AI in order to maintain their trust [12]. Use of AI in mammography raised number of legal implications that need to be carefully considered. One of the main legal implications was the issue of liability. Interviewed women raised the issue of who is legally responsible for errors made by AI reading in screening [20]. Most of the respondents in the Pesapane study considered that both software developers and radiologists are accountable for AI errors [17]. Other legal implications were data privacy, ownership, confidentiality and consent. As per the professionals’ views, data privacy and security and the involvement of large data companies are an important legal issue that need to be brought forward prior to implementation [16].

AI in mammography for early detection of breast cancer may cause a range of social challenges and limitations. Equity and access, trust and acceptance, and the workforce impact were the main social implications identified. As per a study done among Australian women, broader discussions were conducted on acceptance of AI in breast screening; all participants were positive and cared most about AI speeding up the process and getting their results faster [7]. Key concerns addressed by the women were that AI-driven programmes must work effectively and reach all women equally, need to be monitored and preserve equity to ensure equitable access to AI-based healthcare technologies [7]. The impact on the workforce was another social implication that was brought into discussion by the professionals. In Hogberg’s study, nearly half of the breast radiologists believed that there would be a significant impact on their profession [11]. As per the discussions held among professionals, it was emphasised that AI could lead to significant job displacement, training requirements and adaptation for healthcare professionals. However, some study participants considered it a challenge for workforce needs and highlighted the fact of decreased reliance on medical specialists [16].

The cost of AI-driven mammography was one concern raised by participants in Johansson’s study [20]. The Development and implementation cost of AI-driven mammography may be a barrier to adoption in resource-limited settings. In addition, training and education cost, maintenance and updating cost also need to be considered [20]. Translational costs such as diagnostic alignment with laboratory and other services also need to be considered prior to AI being fully embedded into the system as a next-generation strategy. Cost-effectiveness concerns were identified as a challenge; however, robust economic evaluations remain limited in this current literature and acknowledged as a key evidence gap rather than an omission of this review.

#### 2.5.3. Stakeholder Perspectives: Women, Radiologists and PCP Views

Women and radiologists are important stakeholders in clinical practice. Women’s perception and radiologists’ views are important when considering the effectiveness of AI-driven mammography in breast cancer early detection.

Women were generally positive, and they had positive views on AI as a valuable tool in mammography for breast cancer screening due to its potential to improve accuracy and efficiency [7,12,17]. However, women emphasised the importance of radiologists remaining involved in the screening process even with the AI assistance. They were also concerned about transparency, trust and clear communication and education regarding the AI role and limitations of AI [12]. Women expressed their concerns about accountability on AI-related diagnostic errors, and most women preferred the use of an AI adjunct to radiologist judgement [17]. According to an Australian study, screening programmes must ensure that human expertise and responsibility remain central in the programmes; using AI must not alleviate inequities, and programmes need to be monitored and evaluated [7].

Radiologists had a positive view on AI-driven mammography, recognising its potential to improve breast cancer. Increased accuracy, improved efficacy, reduced workload and reduced variability were some potential benefits that professionals discussed [10,11,16]. However, there were concerns on lack of transparency, potential biases and discrepancies, accountability and the impact on their profession [11,16,21].

Most primary healthcare providers accepted the use of AI and radiologist participation in the screening process [10]. According to their overall views, AI has the potential to improve breast cancer screening; however, it is important to address these concerns and ensure these technologies are used safely and effectively.

## 3. Discussion

### 3.1. Benefits of AI-Driven Mammography

Overall, AI-driven mammography has the potential to improve the diagnostic accuracy of breast cancer screening. AI algorithms have diagnostic accuracy and precision superior or comparable to experienced radiologists in breast cancer screening [22]. However, further studies need to evaluate the diagnostic accuracy of AI systems in breast screening [23]. AI-CAD increases the specificity of radiologists without decreasing the sensitivity [4]. It was also identified that replacing one radiologist with AI reading in double reading could be feasible, and that it increases the breast cancer detection accuracy [9]. When evaluating the combined use of AI reading and radiologist assessment to interpret screening mammograms, it was identified that the combined method improved the overall diagnostic accuracy [9].

Replacing one radiologist in double reading with AI reading resulted in increased cancer detection [8]. In a retrospective study done in Korea, to evaluate the performances of screening mammography compared with radiologist and standalone AI, the cancer detection rates and sensitivity were similar between radiologist reading and AI reading; however, they outperformed the radiologists’ reading in relation to specificity, recall rates and positive predictive values [24]. A comparative study done from the data analysis of German, UK and Swedish screening programmes on the use of AI strategy had the largest improvements in breast cancer detection rates and reduction in recall rates [25]. This study highlighted the importance of an increase in breast cancer detection rates by using AI in mammography screening either as a standalone reading or combined reading with a radiologist.

A randomised control trial done in Sweden resulted in significantly lower screening workload by the AI-supported mammography screening when compared to standard double reading by the radiologists [14]. Use of AI-based digital mammography and tomosynthesis could reduce radiologist’s workload up to 70% [18]. In a retrospective study done in the UK to evaluate the effectiveness of AI as a supporting reader for double reading screening, the human workload was substantially reduced by using new AI technology [26]. AI can assist radiologists in identifying the patterns and abnormalities which could be missed by the human eye, increasing the speed and accuracy of interpretation, which results in more efficient workflow [26]. A German study also revealed that the performance of an AI system either as standalone or combined, improves the screening accuracy and reduction in workload of radiologists [27]. An AI support system enhanced the diagnostic performance of radiologists [28]. It is important to identify that AI is intended only to augment the expertise of the radiologists to improve the efficiency of breast cancer screening, not to replace them.

AI assistance significantly reduces the recall rates by reducing false positive rates in breast cancer screening [29]. This will help to reduce the additional costs in the health sector by reviewing false positive cases. A similar study done to determine whether AI-driven mammography reduces unnecessary recalls revealed significant improvement in diagnostic performance and reduction in recall rates [30]. Reduced recall rates would reduce patient anxiety and stress, and, in addition, it would help to lower the healthcare cost by reducing the additional screening and testing.

Interval cancers are more aggressive types of cancers and have poor patient outcomes. AI algorithms have the ability to identify the early development of an interval cancer and to improve the patient outcomes [13]. AI can detect the interval cancers correctly, which could have been missed by human reading. AI can also support radiologists for detecting more cancers, including interval breast cancers [31]. Improvements in risk assessment by AI algorithms could enhance the detection of smaller breast cancers [19]. Developed AI tools showed good performance and were helpful in assessing breast cancer risk prediction [32].

AI-assisted mammography represents a next-generation diagnostic approach that enhances screening accuracy, supports earlier breast cancer detection, reduces radiologist workload, increases interval cancer detection, and holds significant potential to improve population-level screening outcomes through more efficient and equitable breast cancer diagnosis.

### 3.2. Challenges of AI-Driven Mammography

Significant challenges in the use of AI for mammographic screening were biases, discrepancies and accountability issues [11,16]. When an AI algorithm is trained and tested in one setting or a group of population, it may not be applicable to another setting or another set of population, which could create transferability issues [33]. Lack of transparency on AI algorithms used in screening could raise issues on accountability [34]. Transparency was an important measure that was discussed among the women participants [20]. Some of the professionals discussed the lack of insight into ‘black box’ diagnosis and they were worried about the trust in the AI system [16]. An AI system requires vast amounts of data to train and operate, which could raise concerns over patient privacy and data security. Data ownership, confidentiality and consent were other important challenges that need to be addressed [7]. In relation to legal issues and responsibility, who is legally responsible for AI harm is another important implication. Some women suggested that software developers and radiologist are accountable for AI errors [17]. As per Carter, the regulatory gap in clinical application of AI is one significant challenge [7]. It is important to have a clear regulatory framework and a regulatory body to govern the use of AI in healthcare.

Equitable access to AI-driven mammography is one important social implication that was raised by the women in Australia [7]. Public trust and acceptance in the AI system are crucial for successful implementation. Concerns related to biases, privacy, transparency and accountability need to be carefully addressed for the acceptability of AI in breast cancer screening. Impact on the workforce is another important social implication identified in this review. The job displacement of radiologists in the long term is a potential risk, which could arise due to the widespread use of AI in breast cancer screening, and this concern was highlighted in studies conducted among the radiologists [11,16]. However, another study done among radiologists indicated that they did not fear that AI technology will undermine their profession and did not believe that they would be fully replaced by AI [20]. Workforce impact was a considerable social implication of AI-driven mammography; however, effective communication and collaboration of radiologists, AI developers and policymakers would be crucial for a smooth transition and address any potential for workforce impact.

Overall, AI-integrated mammography testing cost could depend on AI implementation cost, training cost and ongoing maintenance cost [35]. Due to out-of-pocket expenses or limited insurance coverage, this could make it difficult for patients to afford enhanced AI screening. Some studies done in the UK revealed the cost-effectiveness of using AI in breast cancer screening programmes [36,37]. The implementation and ongoing maintenance cost of AI in breast cancer screening can be significant; however, the potential for long-term cost-saving and improved patient outcomes makes AI integration for breast cancer screening much more cost-effective.

From a laboratory medicine perspective, integrating AI-driven mammography into existing diagnostic workflows presents additional challenges [25,37]. Effective implementation requires seamless interoperability between radiology systems, laboratory information systems, electronic health records, and pathology-reporting platforms [38]. Standardised data transfer protocols and structured reporting formats are necessary to ensure that AI-generated outputs, such as risk scores, lesion prioritisation, or decision-support flags. Regulatory and validation challenges further complicate laboratory and clinical adoption. AI-based mammography systems are classified as software as a medical device, requiring rigorous regulatory approval and post-market surveillance. Compliance with regulatory frameworks is essential for safe deployment in clinical and laboratory settings. These frameworks require robust clinical validation, transparency of algorithm updates, and ongoing performance monitoring processes that can be resource-intensive and time-consuming.

Addressing the overall legal, ethical, social and implementation challenges, including workflow integration, regulatory compliance, and diagnostic alignment across radiology and laboratory medicine, is crucial for translating AI-assisted mammography from isolated technological innovation into a fully embedded, next-generation diagnostic strategy.

### 3.3. Stakeholder Perception on AI-Driven Mammography

In this review of articles, women generally had a positive view towards the use of AI in breast cancer screening [7,12]. Most of the women agreed on the introduction of AI as an adjunct to the judgement of radiologists, and accountability was an important issue they highlighted [17]. Some women participants were ready to accept the use of AI-driven mammography for breast screening; however, they were not ready to use it for the other health applications. They have mixed views on the issue of trust in AI applications [21]. A study done in Norway found that women had a positive view towards AI use in mammography, but they still needed human involvement in the screening process [39]. Some women preferred radiologists as the first reader and AI as the second reader in mammographic screening, and accountability of AI-driven screening was still a matter to be discussed as a limitation [40]. These findings reveal the mixed views and perceptions of women in implementing AI-driven mammography for breast cancer screening.

Radiologists’ views on integration of AI into mammographic screening were positive, but they emphasised the issues of minimising challenges and significant uncertainties which needed to be addressed before implementing AI into the clinical practice [11,16]. Some Swedish radiologists preferred using AI as a complementary reader rather than an independent reader and they did not feel that their profession would be undermined by using AI [12]. While the radiologists were positive towards AI use in breast cancer screening, there were challenges that they highlighted which needed to be managed prior to fully implementing AI in breast cancer screening. A study done among primary healthcare providers expressed acceptance of integrating AI into breast cancer screening alongside continued radiologist involvement [10,41], viewing this positively as strong support for the implementation of new diagnostic technologies.

From a stakeholder perspective, effective implementation of AI-assisted mammography requires a collaborative, multidisciplinary model that bridges radiology, data science, laboratory medicine, and pathology. This collaborative implementation model positions AI not as an isolated technological intervention, but as an embedded decision-support tool fostering shared accountability, trust, and translational impact across the breast cancer screening continuum.

This discussion synthesises clinical benefits, challenges and stakeholder views, carefully calibrating transitional claims in light of the predominance of reviewing studies related to clinical application of AI-assisted mammography. While promising, AI-assisted mammography requires further prospective, real-world evaluations.

### 3.4. Future Directions: AI-Assisted Mammography Integrated Diagnostic Pathways

To fully realise the translational potential of AI-assisted mammography, future efforts must move beyond standalone performance evaluation towards integrated diagnostic models [41,42]. AI algorithms may identify women at higher risk based on imaging features, enabling targeted follow-up with adjunctive investigations such as ultrasound, Magnetic Resonance Imaging, liquid biopsy, or genomic profiling [37,38]. This layered diagnostic approach has the potential to enhance precision while optimising resource allocation.

Equally important is the development of interdisciplinary training pathways that bridge health sciences, radiology, and data science. A workforce fluent in both clinical reasoning and algorithmic interpretation is essential to ensure safe deployment, appropriate oversight, and meaningful human–AI collaboration. Educational curricula and continuing professional development programmes must evolve to support this shift. Future work will focus on establishing a robust post-deployment monitoring framework for AI-assisted mammography, incorporating continuous performance surveillance in real-world clinical settings, routine detection of performance drift, retraining, and real-time auditing of AI-supported decisions under defined clinical governance and accountability structures.

From a systems perspective, future research should prioritise long-term prospective studies evaluating real-world outcomes, cost-effectiveness, and equity impacts across diverse populations. Regulatory frameworks must evolve in parallel, providing clear guidance on accountability, algorithm updates, data governance, and ethical use. Culturally responsive research exploring acceptability and trust among different communities will be critical to ensure that AI-assisted mammography contributes to equitable breast cancer outcomes globally.

### 3.5. Strengths and Limitations

This narrative review is strengthened by its rigorous methodology, having been conducted in accordance with PRISMA guidelines and critically appraised using the CASP checklist and thematic analysis, supporting its classification as a high-quality review [6,43,44,45,46]. This narrative review includes studies from various countries, which enhanced generalisation through diverse population and geographic representation. A significant strength of this narrative review was its comprehensive synthesis of studies conducted across various countries and among diverse populations. By incorporating research from various national contexts and incorporating populations with different sociodemographic characteristics, it enhances potential generalizability by strengthening the external validity of the arrived conclusions. Including different study methods of research through the integration of qualitative and quantitative studies was another strength identified in this study. This narrative review showed methodological rigour by integrating both quantitative and qualitative research paradigms in a systematic way. This method allowed for a more comprehensive understanding of research topics. The inclusion of studies characterised by large sample sizes constituted a significant strength of this narrative review. Large sample sizes contributed to the reduction in sampling errors, which in turn enhanced the precision and reliability of statistical estimates. By utilising studies with substantial sample sizes, they reinforce the robustness of the evidence base, and the review findings could be interpretated and applied with more confidence.

This review examined insufficient exploration of economic implications and cost-effectiveness. The absence of a thorough cost-effectiveness analysis may limit the practical implications of review findings for healthcare policymakers. Another significant limitation was its disproportionate reliance on studies conducted within high-income countries. This restricts the generalizability of the findings to particularly concerning feasibility and effectiveness of implementing AI in mammography within low- and middle-income countries (LMICs). The unavailability of research from these settings overlooked important factors like resource constraints, infrastructure limitations and variations in healthcare delivery systems. Due to this issue, the applicability of AI-driven mammography to a diverse global context remains uncertain and may need further research specifically tailored to LMIC settings. Neglect of cultural and economic differences was another limitation identified. This review demonstrated a limited consideration of the influence of diverse cultural backgrounds and economic conditions on the implementation of AI technology in mammography for breast cancer screening. This may fail to acknowledge the significant variations in healthcare access, health-seeking behaviour, and socio-economic and socio-cultural norms that can affect the outcomes. Another important limitation of the reviewed studies was the relative short duration of the interventions examined. This limitation could restrict the ability to assess the long-term effects and sustainability of AI-driven mammography. Limitations explicitly include the lack of cost-effectiveness evidence and underrepresentation of LMIC settings which may affect generalisability. To address these limitations, future research should prioritise prospective longitudinal studies with extended follow-up periods.

## 4. Conclusions

This narrative review synthesises a broad body of global evidence on AI-assisted mammography, highlighting its emerging role as a next-generation diagnostic strategy in breast cancer screening. The findings demonstrate that AI-driven mammography has the potential to enhance cancer detection rates, improve diagnostic accuracy and diagnostic efficacy, reduce workload, and improve identification of interval cancers, particularly within diverse populations.

However, the successful integration of AI into clinical practice requires more than technological performance alone. Ethical, legal, social, and economic challenges and implementation challenges remain significant, including issues of transparency, accountability, data governance, integration into the system and equitable access. The perspectives of women and health professionals emphasise the importance of trust, human oversight, workflow compatibility, and professional identity in shaping real-world adoption.

This review proposes that AI-assisted mammography should be embedded within existing radiology and diagnostic workflows as a supportive, rather than substitutive, tool, creating a seamless diagnostic pipeline that integrates human expertise with algorithmic intelligence. Clear regulatory frameworks, robust economic evaluations, and inclusive stakeholder engagement are essential prerequisites for sustainable implementation.

In conclusion, AI-assisted mammography represents a clinically meaningful advancement in breast cancer screening when implemented as a supportive, human-centred diagnostic strategy. Ethical governance, economic evaluation, and equitable access remain essential prerequisites for sustainable adoption. Continued interdisciplinary research, policy development, and stakeholder-centred implementation strategies are required to ensure that AI-driven mammography is deployed ethically, effectively, and equitably, ultimately contributing to improved early detection and reduced global breast cancer mortality.

## Figures and Tables

**Table 1:** Screening questions related to the Critical Appraisal Skills Programme (CASP) tool.

Q No	Screening Question
1	Was there a clear statement or aims of the research?
2	Was the study design appropriate to address the aims of the research?
3	Was the recruitment strategy appropriate to the aims of the research?
4	Was the data collected in a way that addressed the research issue?
5	Were the outcomes measured in a valid manner?
6	Was the data analysis sufficiently rigorous?
7	Have ethical issues been taken into consideration?
8	Was there a clear statement of findings?
9	Were the strengths and limitations addressed clearly?
10	How valuable was the research?

**Table 2:** The summary of literature reviewed.

Article	Study Design and Sample Size	Aims and Objectives	Key Findings	Limitations
1. Carter et al., 2023 [7] (Australia)	Qualitative study; 50 women (8 group discussions)	To understand women’s value and judgement on use of AI for reading mammography in breast cancer screening.	Women saw AI improving accuracy but raised ethical concerns and preferred radiologists to remain central.	Screening outcomes and overdiagnosis not explored.
2. Dembrower et al., 2023 [8] (Sweden)	Prospective clinical trial; 55,581 sample	How AI affects cancer detection.	AI increased detection and reduced recalls, though reliability concerns influenced provider acceptance.	No QA tool and limited generalisability.
3. Elhakim et al., 2023 [9] (Denmark)	Quantitative retrospective study; 158,732 sample	Assess diagnostic accuracy of standalone AI vs. replacing first reader with AI in double reading.	Combined AI–radiologist reading improved accuracy, with concerns about overdiagnosis and cautious clinician acceptance.	Cut-off bias and retrospective design limits generalisability.
4. Hendrix et al., 2021 [10] (USA)	Qualitative study; 91 primary care providers	Explore primary care provider preferences on AI in breast cancer screening.	PCPs valued improved sensitivity but raised safety and ethical concerns and insisted radiologists stay involved.	Limited AI understanding and missing other stakeholder perspectives.
5. Högberg et al., 2023 [11] (Sweden)	Quantitative survey; 105 radiologists	Investigate radiologists’ views on AI-supported mammography screening.	Radiologists saw accuracy benefits but feared legal, ethical and professional risks, leading to cautious acceptance.	Small sample and low response rate.
6. Johansson et al., 2024 [12] (Sweden)	Qualitative study; 16 women	Explore women’s attitudes and perception of AI mammography reading.	Women appreciated accuracy benefits but worried about ethical issues and preferred shared AI–radiologist roles.	Self-report and limited demographic transferability.
7. Kim et al., 2023 [4] (Korea)	Retrospective matched cohort study; 3158 sample	To explore whether AI-CAD improves radiologists’ performance or can be used standalone.	AI-CAD improved specificity and reduced workload, though users preferred supportive use due to reliability concerns.	Small sample and selection bias.
8. Kizildag Yirgin et al., 2022 [13] (Turkey)	Retrospective analysis; 211 sample	Evaluate AI’s diagnostic performance including interval and missed cancers.	AI showed higher accuracy and detected missed cancers, though clinicians noted methodological limitations.	Small sample and no prospective comparison.
9. Lång et al., 2023 [14] (Sweden)	Randomised controlled trial; 80,033 sample	Investigate AI use in mammography for workload reduction and accuracy improvement.	AI increased detection and reduced workload by 44%, though providers remained cautious about implementation risks.	Single-centre and one AI system used.
10. Mansour et al., 2024 [15] (Egypt)	Prospective study; 32,822 sample	Investigate implementing AI in daily screening to reduce workflow.	AI improved detection and workflow efficiency, though clinicians preferred human oversight due to trust concerns.	Single-centre and single vendor limits applicability.
11. Martiniussen et al., 2023 [16] (Norway)	Cross-sectional survey; 98 radiologists	Explore radiologists’ expectations of adding AI to screening interpretation.	Radiologists expected accuracy improvements but highlighted ethical and legal risks influencing cautious acceptance.	Selection bias and small sample.
12. Pesapane et al., 2023 [17] (Italy)	Prospective survey; 870 women	Evaluate women’s knowledge and attitudes on using AI for breast screening.	Women accepted AI as support but rejected full automation due to accountability and error concerns.	High-income setting limits generalisability.
13. Raya-Povedano et al., 2021 [18] (Spain)	Retrospective study; 15,987 sample	Evaluate AI to reduce radiologist workload without reducing cancer detection rate.	AI reduced workload by 70% while maintaining sensitivity, though real-world ethical issues remain unaddressed.	Single vendor and retrospective design.
14. Santeramo et al., 2024 [19] (UK)	Retrospective case–control study; 3386 sample	Evaluate performance of different AI algorithms for detection and risk assessment.	AI improved risk assessment and detection accuracy, though technical and demographic challenges affected applicability.	Retrospective design and limited machine types.

**Table 3:** Themes and subthemes.

	Themes	Subthemes
1.	Benefits	Increased Diagnostic accuracy Increased Diagnostic efficacy Enhanced cancer detection rates Improved Interval cancer detection
2.	Challenges	Ethical implications Legal implications Social implications Implementational challenges
3.	Stakeholder perception	Women’s views Radiologists’ views Primary Care Providers (PCP) views

## Data Availability

No new data were created or analysed in this study. Data sharing is not applicable to this article.

## Abbreviations

AI	Artificial Intelligence
AI CAD	Artificial Intelligence Computer-Aided Diagnosis
CASP	Critical Appraisal Skills Programme
LMIC	Lower Middle-Income Countries
MRI	Magnetic Resonance Imaging
PCP	Primary Care Providers
PRISMA	Preferred Reporting Items for Systematic Reviews and Meta Analysis
USS	Ultrasound Scan
